# Early results of a prospective study on the pyrolytic carbon (pyrocarbon) Amandys® for osteoarthritis of the wrist

**DOI:** 10.1308/003588412X13373405386655

**Published:** 2012-10

**Authors:** ZJ Daruwalla, KL Davies, A Shafighian, NR Gillham

**Affiliations:** ^1^Oxford University Hospitals NHS Trust,UK; ^2^Horton Treatment Centre,UK

**Keywords:** Amandys®, Pyrolytic carbon, Scapholunate advanced collapse, Wrist osteoarthritis

## Abstract

**INTRODUCTION:**

The preliminary results of a pyrocarbon interpositional radiocarpal implant in a small cohort of patients were reviewed. As it is currently only a limited release product, we describe to potential users early complications and negative outcomes.

**METHODS:**

Patients were assessed using pain levels, ranges of motion, grip strength, type of and time to return to work as well as pre-operative and post-operative DASH (Disabilities of the Arm, Shoulder and Hand) scores. Radiographs were taken and patient satisfaction was recorded.

**RESULTS:**

All six patients were contacted. One was not satisfied. Three had reduced motion. None experienced squeaking. There were no immediate or late post-operative complications. There was one early volar displacement of an implant.

**CONCLUSIONS:**

Although our early results are somewhat encouraging, further and longer studies are warranted before supporting the use of this particular pyrocarbon implant as a primary procedure.

Scapholunate advanced collapse (SLAC) and osteoarthritis of the wrist are well recognised conditions that are considered very difficult to treat, not only by general orthopaedic surgeons but also by hand and upper limb specialists. Some of the more popular treatment options for SLAC wrists include proximal row carpectomy (PRC) and four corner fusion (4CF).

A systematic review in 2009 compared these two procedures and found grip strength, pain relief and subjective outcomes to be similar in both treatment groups.^1^ The same review also found that PRC provides a better post-operative range of movement and lacks the potential complications specific to 4CF such as non-union, hardware issues and dorsal impingement although it has a significantly higher risk of subsequent osteoarthritis. Regardless of the procedure performed, both have their own specific associated complications and see progression of disease with a significant proportion of patients needing further, technically difficult, major procedures, usually in the form of total wrist fusion, which in turn reduces function by abolishing wrist flexion and extension.

With regard to osteoarthritis of the wrist, a variety of surgical options also exists including arthroscopic wrist procedures, denervation, synovectomy, ulnar resection or replacement, arthrodesis and arthroplasty, depending on the extent of the arthritis.^2^ Again, regardless of procedure performed, all have their own specific associated complications. In an article from 2011 on mid-term follow-up of universal total wrist arthroplasties, the authors reported 5 complications in their series of 21 patients.^3^

With such complication rates as well as the issues mentioned previously, it is difficult to offer such procedures to patients, and expect excellent results and patient satisfaction. Indeed, if PRC or 4CF were considered as joint replacements are, current published results would not be acceptable. An alternative procedure in cases of SLAC wrists is to just resect part or all of the scaphoid. This, however, results in collapse of the carpus and necessitates a further procedure. Previous attempts to replace the scaphoid have failed, primarily due to the material being too soft (silastic),^4^ too hard (metal or ceramic) or causing severe synovitis.^5^ A replacement with a material with a Young’s modulus close to bone may have better results. Such an option exists with pyrolytic carbon (pyrocarbon).

While the Adaptive Proximal Scaphoid Implant (APSI) (Tornier, Montbonnot, France) is a non-fixed orthopaedic implant designed to replace the proximal pole of the scaphoid, the Amandys® (Fig 1) (Tornier) is a non-fixed, olive shaped pyrocarbon interpositional implant for replacement of the wrist joint that maintains carpal coherence and preserves the ‘dart throwing’ action of the wrist.^6^ With no published studies on the Amandys® and only two studies on the APSI showing promising results,^4,7^ our paper presents the early results of a prospective study on pyrocarbon interpositional arthroplasty as an alternative treatment option for osteoarthritis of the wrist and SLAC with the use of the Amandys® as first described and presented by Bellemere *et al*.^8,9^ As this implant is currently only a limited release product, this paper further describes to potential users the early complications and negative outcomes we have experienced.

**Figure 1:**
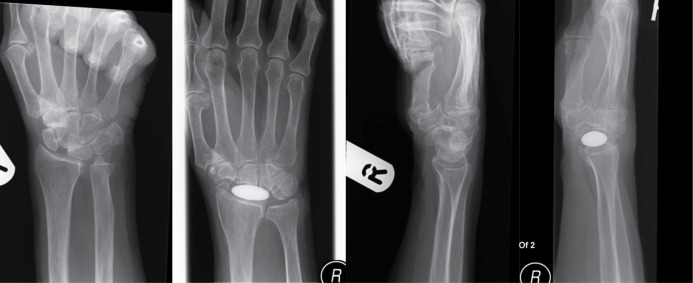
Post-operative radiographs of a patient with an Amandys® for osteoarthritis of the wrist and scapholunate advanced collapse

## Methods

This study followed six consecutive Amandys® arthroplasty day cases prospectively between 2009 and 2011. Institutional review board approval and informed patient consent were both obtained. Exclusion criteria for day case surgery included patients being deemed medically unfit, travel time exceeding one hour, not having a responsible adult escorting the patient home and supervising them overnight as well as patients having a body mass index of >40kg/m^2^. There were two men and four women. The mean patient age was 55 years (range: 42–67 years). The dominant hand was involved in five patients. Patients attended for pre-assessment on the same day as their outpatient appointment and were given information regarding the procedure, day case protocol and the post-operative physiotherapy regime. All procedures were performed under general anaesthesia and by the senior author, with tourniquet times recorded.

A routine dorsal approach to the wrist between the third and fourth compartments is used. Additional mobilisation of the second compartment is also undertaken. This provides access to the proximal scaphoid and the radial styloid as well as the lunate. The bony resection for the Amandys® consists of a styloidectomy, the proximal half of the scaphoid, all the lunate and a wafer from the capitate. A little more than the capitate’s articular surface is resected to expose subchondral bone. This is further reamed with an egg shaped burr to create a concavity complementing the convexity of the implant’s distal surface. A jig is supplied to correctly orientate the trial because its proximal surface is slightly more curved to match the anatomical concavity of the distal radial surface.

A styloidectomy is carried out to allow the implant to be accommodated with the wrist in neutral and to permit a certain amount of radial deviation that is not limited prematurely by styloscaphoid impingement. No more than 7mm in height should be excised as this risks detaching the extrinsic ligaments of the wrist. In addition, the floor of the first extensor compartment must not be perforated during removal of the styloid as this may provide a path for escape of what is an unconstrained implant.

Any defect must be repaired securely along with the dorsal wrist capsule at the end of the procedure. Similarly, removal of the lunate must be performed carefully so as not to violate the volar extrinsic ligaments of the wrist. These, if damaged, must also be repaired to prevent a potential volar escape of the implant into the carpal tunnel. We recommend incomplete resection of the lunate leaving behind a sliver of it still attached to the volar capsule. In this way, one may be certain that the ligaments have not been violated and the implant is still accommodated with ease.

Trial implants of varying size are tested under fluoroscopic control for stability. The correct size is one that allows a full range of motion without dislocating at the extremes. An implant that is ‘stable on the table’ will remain so in the long term once the soft tissues have healed.

Rehabilitation commenced with a physiotherapy regime three weeks post-operatively, to allow time for encapsulation of the prosthesis prior to mobilisation. Prior to this, patients were fitted with Futuro® splints (3M, Bracknell, UK; removable wrist supports with mouldable metal inserts and hook-and-loop fasteners) that were kept on at all times, only allowing finger mobility. At three weeks, in order to prevent stiffness and increase the range of motion as well as reduce swelling and regain finger function, exercises consisted of gentle active and active assisted wrist movement, within the patient’s comfort level only.

Six weeks post-operatively, once the senior author was satisfied with the implant position radiographically, the Futuro® splint was weaned off with the patient advised to use his or her hand for light activities of daily living such as dressing and preparing meals. At that stage, the aims of treatment were to further increase range of motion, build grip strength and strengthen the biceps, triceps, brachioradialis and any areas of weakness arising from the shoulder stabilisers.

Although the wrist had been splinted for only six weeks, overall arm function is still decreased. In order to address this and treat the arm as a complete functional unit, it is vital to address all the above mentioned muscles to achieve full functional rehabilitation. As wrist movement improved and functional movement was achieved, strengthening of the wrist was progressed with particular importance placed on strengthening the extensor carpi ulnaris in supination for stability. Patients were followed up for three months following surgery with the goals of achieving adequate wrist range of motion and grip strength for good functional use of the hand and wrist. Further improvement over the subsequent nine months was expected and this was explained to the patients.

Patients were assessed by a single physiotherapist using level of pain, ranges of motion, type of and time to return to work and DASH (Disabilities of the Arm, Shoulder and Hand) scores, both pre-operatively as well as between 8 and 28 months post-operatively with a mean of 16 months. Using a two-sample t-test, data were analysed to establish whether the differences between the mean values of the pain and DASH scores were statistically significant. This was performed with a 5% significance level (*p*<0.05) and the variance of each sample was assumed to be equal. At the final follow-up appointment, radiographs were taken and assessed by a consultant radiologist with an interest in musculoskeletal radiology. Patients were also asked by a member of staff not involved in the case whether they were satisfied with their procedure.

## Results

All six patients could be contacted. Four cases were of SLAC, one of osteoarthritis of the wrist and one of proximal carpal row destruction in a patient with rheumatoid arthritis. Three patients were satisfied and two very satisfied with their surgery. Only one patient was not satisfied. In one of the patients who was very satisfied, the Amandys® implant was found to be volar displaced one month post-operatively. The implant was relocated, the capsule repaired and a soft tissue release performed with the patient ultimately achieving an excellent result. No patients experienced squeaking. While our study initially had seven patients, one was only eight weeks since insertion of her Amandys® so was not included in the study. This patient required an Amandys® secondary to a traumatic volar displacement of an APSI and conversion from scaphoid non-union advanced collapse to SLAC.

**Figure 2:**
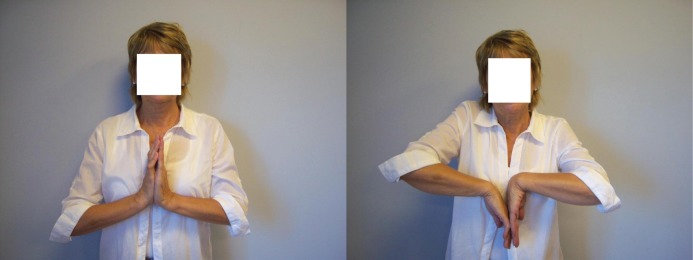
Post-operative clinical photographs of the worst Amandys® case in our study, the patient complaining of a reduced active range of motion compared with before surgery

The only patient not satisfied with the surgery was a 67-year-old woman (Fig 2). While her visual analogue pain score improved from 86 to 34, her DASH score was worse at the final follow-up appointment (41 vs 35).

Pain scores as measured on a visual analogue scale (0–100) were found to be improved with a mean score of 33 (range: 0–67) post-operatively versus 82 (range: 50–90) pre-operatively (*p*<0.05). Three patients had better ranges of motion in terms of wrist flexion and extension as well as radial and ulnar deviation. Compared with the contralateral side, wrist flexion and extension and radial and ulnar deviation were 62%, 73%, 92% and 62% respectively. A full range of pronation and supination remained unchanged except for in one patient who had no supination pre-operatively or post-operatively. With all patients stating their grip was weaker pre-operatively, the mean post-operative gross grip strength was 14kg (range: 0–36kg), correlating to 58% of the contralateral hand. Mean thumb pinch, thumb key and thumb tripod strengths were 3.5kg (range: 0–10.5kg), 5kg (range: 1–11.5kg) and 4.5kg (range: 0.5–10.5kg), correlating to 76%, 65% and 78% of the contralateral hand respectively.

Improved DASH scores were noted in all patients except one with a mean of 29 (range: 5–67) post-operatively compared with 55 (range: 26–89) pre-operatively (*p*=0.07). The specific DASH work and sports/performing arts modules also showed improvements with a mean of 19 (range: 6–25) and 50 post-operatively respectively compared with 53 (range: 25–75) and 75 pre-operatively (*p*=0.03, *p*=N/A as only one patient).

The mean time to return to work was 8 weeks (range: 0.5–16 weeks). The mean follow-up duration was 16 months (range: 8–28 months). Table 1 shows a summary of the results. All post-operative radiographs at the time of the final follow-up appointment noted excellent position of the implants and showed no progressive pathology.

**Table 1:** Mean visual analogue pain and DASH (Disabilities of the Arm, Shoulder and Hand) scores, lengths of return to work, ranges of pre and post-operative active motion at final follow-up appointment and strengths of gross grip, thumb pinch, thumb key and thumb tripod

	Pre-operatively	Post-operatively	% of contralateral
Pain	82	33	–
DASH	55	29	–
DASH work module	53	19	–
DASH sports/performing arts module	75	50	–
Time to return to work (weeks)	–	8	–
Flexion (degrees)	40	40	62
Extension (degrees)	39	42	73
Radial deviation (degrees)	18	12	92
Ulnar deviation (degrees)	23	17	62
Pronation (degrees)	90	90	100
Supination (degrees)	75	75	100
Gross grip (kg)	–	14	58
Thumb pinch (kg)	–	3.5	76
Thumb key (kg)	–	5	65
Thumb tripod (kg)	–	4.5	78

## Discussion

Despite advances in orthopaedic surgery, surgical options for SLAC and osteoarthritis of the wrist continue to raise debate as to what is best for the patient although validated algorithms for the treatment of SLAC do exist. Salvage procedures like PRC and 4CF are two of the more popular operations but both have drawbacks, including loss of grip strength and movement respectively. Another procedure, namely wrist arthrodesis, while surprisingly still considered the ultimate solution for post-traumatic wrist osteoarthritis,^10^ unsurprisingly still results in poor hand function.^11^ While many patients scheduled for these procedures are demanding in respect to the function they need in the wrist to enable them to return to work and leisure activities, this paper challenges the assumption that there are no surgical alternatives, and questions the promotion and use of the Amandys®.

Being thromboresistant,^4^ pyrocarbon remains the most widely used material for mechanical heart valves and was first introduced to the medical industry by chance in the 1960s after artificial heart valves constructed from other materials failed secondary to blood clotting.^12^ With the ideal orthopaedic implant having characteristics that match those of bone, the Amandys® takes advantage of being manufactured from pyrocarbon. Not only does this make the implant thromboresistant but also biocompatible, biochemically inert, highly wear resistant and, most importantly, very similar to cortical bone (same elastic modulus and density), the latter resulting in reduced chances of stress shielding.^13^ However, its high cost may be a limiting factor in its use as the implant costs between £1,500 and £2,500.

While the use of pyrocarbon implants in orthopaedic surgery is not novel,^14–16^ no published studies on the Amandys® exist. Although one patient required a secondary procedure, this does not represent a failure of the initial procedure with the patient herself satisfied. This patient was the first on whom the senior author performed an Amandys® arthroplasty and allowed to mobilise gently immediately following the operation. Within a month, volar displacement of the Amandys® occurred. During its relocation, the capsule was noted to be deficient on the volar aspect, and this was thought to be due to too early and excessive mobilisation.

As a result, the senior author now takes a degree of caution immediately following surgery with regard to the level of mobilisation and heavy work that the patient is allowed with this implant in situ. It should be noted, however, that some patients are non-compliant with the recommended physiotherapy regime but still demonstrate excellent outcomes. For example, one patient, a 42-year-old male plasterer, returned to work within four days. At his second last follow-up visit (eight months since surgery) he stated that he had been carrying loads of up to 80kg within weeks of his operation.

The only patient in our study not satisfied with the surgery was a 67-year-old woman. This patient was the second on whom the senior author performed an Amandys® arthroplasty, a month after the previously mentioned case with volar displacement of the implant. While her visual analogue pain score improved from 86 to 34, her DASH score was found to be worse at the final follow-up appointment (41 vs 35).

This patient was also one of three who had a reduced post-operative active range of motion. The senior author attributes this to overcautious tightening and closure of the capsule and soft tissue as well as prolonged immobilisation due to the above mentioned post-operative complication from his first case, both demonstrating the steep learning curve associated with the Amandys®. While this was unfortunate, it was explained to the patient that the alternative option of arthrodesis would have resulted in complete loss of movement and that perhaps this result should not be considered unsatisfactory considering the significant improvement of pain. With regard to the other two patients with reduced active ranges of motion, one of them was the patient described previously to have had the dislocated Amandys® relocated while the other had very debilitating rheumatoid arthritis.

The Amandys® offers quick and minimally invasive surgery, good pain relief and a speedy return to a ‘normal wrist’. In sole comparison with arthrodesis, although three patients had worse active ranges of motion post-operatively, an Amandys® is certainly a more viable and attractive option than a complete abolishment of wrist motion.

In terms of functional range of wrist motion, a summary of PRC and 4CF results shows an average of 37° and 33° of flexion, 41° and 34° of extension and a grip strength 78% and 74% of the contralateral side respectively.^17^ Comparing just these two procedures with the Amandys®, our study shows a similar range of motion with 40° of flexion, 42° of extension and a grip strength 58%, 76%, 65% and 78% of the contralateral side in terms of gross grip, thumb pinch, thumb key and thumb tripod strengths respectively. Grip strength and key pinch were only assessed post-operatively due to the pre-operative inability to perform the test because of pain.

## Conclusions

Should the prosthesis fail, then alternative salvage options such as arthrodesis and arthroplasty are not compromised as there is no evidence to suggest any of these salvage procedures would be more difficult following Amandys® arthroplasty. With our current results, the senior author is of the opinion that there is no rationale for a major ablative procedure when an easy alternative offers the potential for better function. Although wrist arthroplasty may result in better post-operative active range of motion, in cases where PRC, 4CF or arthrodesis are considered, we propose that the Amandys® may be an alternative option.

There are no long-term data for pyrocarbon interpositional arthroplasty of the hand. With the timescale of recovery known to be lengthy and often extending beyond a year following the previously mentioned salvage procedures, our early results are encouraging and support the use of the Amandys® as an alternative treatment option for osteoarthritis of the wrist and SLAC. However, with one recent study bringing to light long-term concerns about pyrocarbon and its interface with bone,^18^ and another suggesting that pyrocarbon interposition does not significantly improve post-operative function and carries a high post-operative risk of pyrocarbon displacement and need for revision surgery,^19^ our early results warrant further and longer studies.

With the Amandys® burning no bridges for further surgery, any surgeon considering using this implant as a first procedure in what is a generally young and active subgroup of patients should be aware of both, the promising results as well as the complications and negative outcomes outlined in this paper.
